# CCN Family Proteins in Cancer: Insight Into Their Structures and Coordination Role in Tumor Microenvironment

**DOI:** 10.3389/fgene.2021.649387

**Published:** 2021-03-23

**Authors:** Qingan Jia, Binghui Xu, Yaoyao Zhang, Arshad Ali, Xia Liao

**Affiliations:** ^1^Institute of Medical Research, Northwestern Polytechnical University, Xi’an, China; ^2^School of Life Sciences, Northwestern Polytechnical University, Xi’an, China; ^3^Department of Nutrition, First Affiliated Hospital of Xi’an Jiaotong University, Xi’an, China

**Keywords:** CCN proteins, isoforms, targeted therapy, tumor microenvironment, pan-cancer

## Abstract

The crosstalk between tumor cells and the tumor microenvironment (TME), triggers a variety of critical signaling pathways and promotes the malignant progression of cancer. The success rate of cancer therapy through targeting single molecule of this crosstalk may be extremely low, whereas co-targeting multiple components could be complicated design and likely to have more side effects. The six members of cellular communication network (CCN) family proteins are scaffolding proteins that may govern the TME, and several studies have shown targeted therapy of CCN family proteins may be effective for the treatment of cancer. CCN protein family shares similar structures, and they mutually reinforce and neutralize each other to serve various roles that are tightly regulated in a spatiotemporal manner by the TME. Here, we review the current knowledge on the structures and roles of CCN proteins in different types of cancer. We also analyze CCN mRNA expression, and reasons for its diverse relationship to prognosis in different cancers. In this review, we conclude that the discrepant functions of CCN proteins in different types of cancer are attributed to diverse TME and CCN truncated isoforms, and speculate that targeting CCN proteins to rebalance the TME could be a potent anti-cancer strategy.

## Introduction

Cancer is the second leading cause of death in the United States and is becoming a major public health problem and central focus of modern medical research in China (Arbyn et al., 2020). Although early diagnosis and surgical resection are primary anti-tumor strategies, the prognosis of cancer patients remain generally dismal, with unfavorable outcomes attributed to the high frequency of tumor recurrence, metastasis and therapeutic resistance (Winkler et al., 2020). Therefore, continued identification of new molecules for the development of molecular targeted therapy is still urgently needed (Jiang et al., 2019). An increasing body of research suggests that crosstalk between tumor cells and the tumor microenvironment (TME), including revascularization, immune tolerance, fibrotic components and many cytokines, trigger a variety of critical signaling pathways and promotes the malignant progression of cancer in an integrated manner. Thus, the efficacy of targeting single molecule in cancer therapy may be low, whereas combination therapy could be more benefit for human cancers (Palmer and Sorger, 2017). Here, we present a scaffolding-like protein family that can bind with a variety of molecules and exhibit a multi-target regulatory effects through orchestrating the TME and intracellular signaling pathways.

Cellular communication network (CCN) family are scaffolding proteins that may govern and balance the interconnection among individual signaling pathways. CCN proteins, first described in 1993, are a six-member family of cysteine-rich regulatory proteins that exist only in vertebrates, including CCN1 (cysteine-rich 61, CYR61), CCN2 (connective tissue growth factor, CTGF), CCN3 (nephroblastoma overexpressed, NOV), CCN4 (Wnt1-inducible signaling pathway proteins, WISP-1), CCN5 (WISP-2), and CCN6 (WISP-3). CCN proteins do not behave like individual cytokines in that they do not perform a single function but instead coordinate in various functions of extracellular and intracellular proteins (Perbal, 2018). All CCN proteins serve as extracellular, cytoplasmic and nuclear proteins in their full-length and/or truncated forms and play key roles in regulating tumor cellular function and crosstalk with the TME (Brigstock, 2003). Thus, targeting CCN proteins expression hold promise for remodeling the TME and rebalancing intracellular signaling pathways (Jun and Lau, 2011; Jia et al., 2016).

Although CCN proteins were discovered three decades ago, they have not received widespread interest, and their roles and modes of action in human cancers are still ambiguous. CCN protein members always appear to have paradoxical effects across different types of cancer (Li et al., 2016) and even within the same cancer (Kleer, 2016), and which were often due to the diverse TME. Thus, summative work and further investigations are urgently needed to dissect the actions of CCN proteins considering the diverse TME and their multifunctional domains. Here, we review the current knowledge on the structures and roles of CCN proteins in different types of cancer. We also analyze CCN mRNA expression, its relationship to prognosis, and its isoforms in pan-cancer based on The Cancer Genome Atlas (TCGA) using the bioinformatics tool GEPIA2 (Tang et al., 2019). We conclude that the contradictory nature of the biological properties of CCN proteins in cancer are attributed to their multiple functional domains, which allow them to act as multifunctional regulators in the TME and cancer signaling pathways, and speculate that targeting CCN proteins could be a potent anti-cancer strategy, and the efficacy of which is orchestrated by the different location and existence of diverse ligands.

## Structures and Functions of Full-Length CCN Proteins in Cancer

**FIGURE 1:**
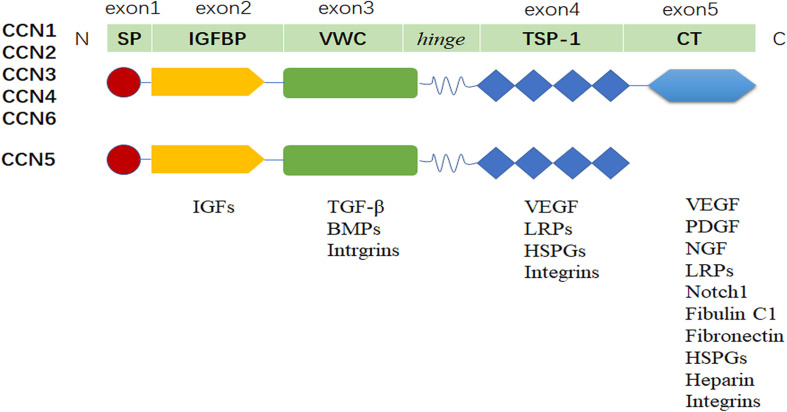
Structure of CCN proteins. Schematic of four conserved functional domains coded by associated exons. CCN proteins could serve different functions via interactions with a variety of cell surface receptors and extracellular ligands (e.g., integrins, HSPGs, LRPs, TGFβ, VEGF, and PDGF).

CCN proteins are secreted proteins, with full-length CCN proteins consisting of a signal peptide for extracellular release followed by four structural domains (with CCN5 lacking the CT domain): IGFBP, VWC, TSP-1, and CT (Perbal and Perbal, 2016). Prototypic CCN proteins are encoded by five exons. Exon 1 encodes a signal peptide, and exons 2– 5 encode IGFBP, VWC, TSP-1, and CT modules, respectively. CCN proteins exhibit similar structure with 60% amino acid homology, and share a series of 38 cysteine residues that are strictly conserved in position and number. Owing to the signal peptide, CCN proteins are characteristically expressed in the cytoplasm and accumulate in the external environment in the form of paracrine. Their four discrete functional domains determine the types of binding ligands with which they interact, including diverse integrins, HSPGs, IGFs, TGFβ, VEGF, and LRPs et al., resulting in a variety of biological functions of full-length CCN proteins (Perbal, 2004; Figure 1).

CCN proteins are multifunctional regulatory molecules in the TME that are involved in many vital biological functions, including angiogenesis, fibrosis, tissue regeneration and repair, and cancer (Yeger and Perbal, 2016). The diverse functions of CCN proteins in the TME are attributed to their modular structural features, which allow binding and interactions with well-known functional ligands (Holbourn et al., 2009). CCN proteins also serve as cytoplasmic and nuclear proteins in their truncated forms and play key roles in regulating tumor cellular function. Thereof, CCN proteins physically located at the center of communication network and exhibit diverse functionalities (Perbal, 2019).

### Multi-Domain Structure of Full-Length CCN Proteins

#### IGFBP Domain in CCN Proteins

The insulin-like growth factor-binding protein (IGFBP) domain of CCN proteins is found in every CCN family members, and shares strong sequence homology to the N-terminal domain of traditional IGFBPs, which bind to and influence the actions of IGFs (Perbal, 2018). Although its IGF binding ability is lower than that of full-length IGFBPs, the IGFBP domain of CCN3 reduces activation of IGF1-IGF1R signaling in inflammatory breast cancer, and downregulation of CCN6 enhances the effects of IGF1 on growth, motility, and invasiveness (Kleer et al., 2004; Zhang et al., 2005). Repudi et al. (2013) reported that CCN6 not only co-localizes with IGF1 but also blocks IGF1 secretion. Different CCN family members exhibit diverse IGF binding ability, in CCN3, the IGFBP domain cannot substitutes for the IGFBP3 amino-proximal sequence for IGF binding (Yan et al., 2006). Up to now, little information is available concerning the exact roles played by the IGFBP domain in CCN function, but the direct and indirect control of IGF function implicates CCN proteins could be a promising intervention strategy.

#### VWC Domain in CCN Proteins

The Von Willebrand factor type C (VWC) domain is also found in every CCN family member, and the VWC domain most commonly binds to bone morphogenic proteins (BMPs) (Canalis, 2007), TGF-β (Inkson et al., 2008), and diverse integrins (i.e., αMβ2, α2β1, αvβ5, α5β1, α6β1) (Kaur and Roberts, 2021). In CCN2, its interaction with TGF-β enhances TGF-β signaling, such that CCN2 might function as a chaperone for TGF-β, and less TGF-β is required to stimulate downstream signaling (Abreu et al., 2002). In CCN3, its interaction with BMP2 inhibits BMP2-induced osteoblast differentiation (Minamizato et al., 2007). Integrins, the primary signaling receptors of CCN proteins, consist of α- and β-subunits that are commonly transmembrane (Karimi et al., 2018). The VWC domain in CCN proteins binds with various integrin subtypes that differ across CCN family members, thereby mediating different forms of cell adhesion and activating signaling pathways in tumor and stromal cells (Li et al., 2015). The ability of the VWC domain to bind with functional ligands suggests that it plays a key role in some biological functions associated with CCN proteins. In considering the interactions between the VWC domain in CCN proteins and TGF-β, BMP-4 et al., the CCN proteins could also be a potential target for cancer therapy, while the specific roles are depended on the type and number of ligands in the TME.

#### TSP-1 Domain in CCN Proteins

The thrombospondin type 1 repeat (TSP-1) domain is another common domain in CCN proteins and plays strong roles in some biological functions of tumor, primarily through interactions with lipoprotein-related receptors (Gerritsen et al., 2016), vascular endothelial growth factor (VEGF) (Tsai et al., 2017), diverse integrins (Alday-Parejo et al., 2019), and heparan sulfate proteoglycans (HSPGs) (Neubauer et al., 2017). As the TSP-1 domain is conserved across CCN family members, this suggests that all CCN family members modulate cell adhesion, maintains ECM composition, and participates in regulating tumor signaling (Jayakumar et al., 2017). Indeed, some studies have linked CCN proteins with mutant or missing TSP-1 domains with colorectal and gastric carcinomas (Perbal, 2016) and Wilm’s tumors (Subramaniam et al., 2008). Therefore, the TSP-1 domain, like other CCN domains, could be a potential target of cancer therapy (Leask, 2020).

#### CT Domain in CCN Proteins

The carboxyterminal (CT) domain is thought to mediate key functions in several CCN proteins (except CCN5), because it also acts as a dimerization module in a manner analogous to domains in other molecules, such as nerve growth factor (NGF), TGF-β, VEGF, BMPs, platelet-derived growth factor (PDGF) and diverse integrins. In addition, many biological functions of cytokines arise through their interactions with heparin (Crijns et al., 2020). Interestingly, many basic residues at the CT domain in N-terminus follow the heparin-binding pattern, suggesting heparin as a candidate for CCN protein-targeted therapy (Jia S. et al., 2017). Its interactions with Notch, lipoprotein receptor-related protein 6 (LRP6), and integrin α6β1 suggest that CCN proteins regulate cellular differentiation and proliferation (Thakur and Mishra, 2016). Furthermore, CT domain-mediated dimerization likely influences other domains in CCN proteins, such as VWC domain (Perbal, 2006b). Together, these reports indicate that the CT domain of CCN proteins plays a crucial role in regulating tumor biology.

### Functions and Progress of Full-Length CCN Proteins in Tumor Progression

#### CCN Proteins Acting as Critical Modulators of the TME

One fascinating aspect of TME that adds to the complexity of tumor progression. CCN proteins can be potential therapeutic targets that can be manipulated to rebalance the TME. Recently, Tao et al. proved that CCN4 was preferentially secreted by glioma stem cells (GSCs), and which played critical roles in maintaining GSCs and tumor-supportive macrophage (Tao et al., 2020). Jia et al. also proved CCN4-induced type I collagen linearization facilitates tumor cell invasion and promotes spontaneous breast cancer metastasis, without significantly affecting gene expression (Jia et al., 2019). CCN2 and its fragments also have been implicated in the regulation of a multitude of biological phenomena in cancers, which was not only associated with fibrosis, but also with mesenchymal stem cells (Leguit et al., 2021). Different CCN proteins also enhance or suppress each other’s action in the TME (Peidl et al., 2019). The available evidence strongly supports that CCN proteins are related to the tumor progression, while the same CCN proteins play different roles in the same type of cancer, and the reason is related to the complexity of the TME (Li et al., 2015; Yeger and Perbal, 2016). Based on these, the final biological properties of the CCN proteins might be dependent on different combinations, and the cocktail containing CCN proteins in different combinations should be applied to rebalance the TME in tumor therapy.

#### CCN Proteins Acting as Direct Modulators of Tumor Progression

Recently, CCN members also play direct roles in tumor progression through diverse signaling pathways. CCN1 has been shown to promotes cell adhesion and migration as a mediator of Notch1 signaling in breast cancer (Ilhan et al., 2020). Overexpression of CCN2 also has been shown to induce the upregulation expression of Wnt/β-catenin transcriptional target genes, and our group also proved CCN2 was associated with the Wnt signaling activation in hepatocellular carcinoma (HCC) (Jia S. et al., 2017). CCN3 has been proved to promotes epithelial-mesenchymal transition (EMT) via FAK/Akt/HIF-1α/twist signaling in prostate cancer (Chen et al., 2017). CCN4 also has been proved to stimulates melanoma invasion and metastasis by promoting EMT-like process (Deng et al., 2019). CCN5 is a tumor suppressor, which restored ER-α expression at the transcription level via integrins-α6β1/Akt/FOXO3a signaling activation in breast cancer (Sarkar et al., 2017). CCN6 is also acts as a tumor suppressor in HCC by negative regulation of β-catenin/TCF/LEF signaling (Gao et al., 2019). Because of the four functional domains of CCN proteins, CCNs mediate tumor progression primarily through binding and interacting with well-known receptors, including integrins, HSPGs, IGFs and LRPs relating the signaling pathways such as Wnts, TGF-β, and Notch signaling et al. (Li et al., 2015).

### Functions and Progress of Truncated CCNs Associated With Cancer Progression

**FIGURE 2:**
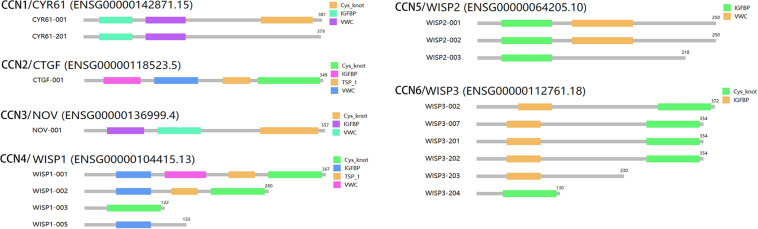
Isoform structures of CCN family members were obtained from GEPIA2 based on the TCGA/GTEx data. Truncated isoforms of different CCN family members indicate diverse biological functions with based on their different functional domains.

CCN proteins lacking one or more of the functional domains can be produced by alternative splicing (Perbal, 2009) or post-translational processing (Viloria and Hill, 2016). The existence of CCN isoforms may have different activities than full-length CCNs and may be regarded as a means of increasing the diversity of their biological roles in cancer (Kaasboll et al., 2018). Our GEPIA2 analysis provides the schematic organization of various CCN isoforms (Figure 2). Despite compelling evidence of the important biological activities of these CCN isoforms, their potential regulatory functions are still vague. Truncated CCN proteins deprived of a signal peptide commonly exist in cytoplasm and/or nucleus have been identified in several physiology and pathological situations (Perbal, 2006a; Planque et al., 2006). Nuclear localization of truncated CCN proteins could serve as a transcriptional factors. Also, their nuclear localization could be influenced by their CT domain (Bleau et al., 2007). Therefore, the existence of truncated CCN proteins could be an important means to discovering their diverse biological functions in different types of cancer. However, the intracellular localization and diverse function of truncated forms of CCN proteins are still unclear and has been a primary research focus of our group.

## Diverse Expression and Roles of CCN Family Members in Pan-Cancer

Although all CCN family members (except CCN5) have four highly conserved functional domains, they have different roles within particular types of cancer. Some CCN proteins have established associations with cancer malignancy progression and are considered as prognostic markers and therapeutic targets for certain types of cancer (Jun and Lau, 2011). However, CCN proteins always appear to have contradictory roles in different types of cancer, which may be due to differences in their TMEs and isoforms (Peidl et al., 2019; Figure 2 and Table 1).

**TABLE 1:** Roles of CCN1-6 in pan-cancer.

**Cancer type**	**CCN1**	**CCN2**	**CCN3**	**CCN4**	**CCN5**	**CCN6**
Bladder cancer	↑(Khandelwal et al., 2020)	↑(Wang et al., 2017)		↑(Lee et al., 2018)		↑(Zeng et al., 2015)
Breast cancer	↑(Xie et al., 2001a)	↑(Shimo et al., 2006)	↓(Dobson et al., 2014)	↑(Xie et al., 2001b; Chiang et al., 2015) ↓(Taghavi et al., 2016)	↑(Zoubine et al., 2001; Banerjee et al., 2003) ↓(Haque et al., 2015)	↓(Martin et al., 2017)
Chondrosarcoma	↑(Tan et al., 2009)			↑(Hou et al., 2011)		↑(Fong et al., 2012)
Colorectal tumor	↑(Jeong et al., 2014; Xie et al., 2019)	↑(Ubink et al., 2016)	↓(Li et al., 2017)	↑(Fischer et al., 2001; Wu et al., 2016)	↓(Davies et al., 2010)	↓(Lu et al., 2016)
Esophageal cancer	↑(Xie et al., 2011) ↓(Dang et al., 2017, 2018)	↑(Deng et al., 2007)		↑(Nagai et al., 2011)	↓(Chai et al., 2019)	
Gastric cancer	↑(Mao et al., 2011; Su et al., 2019)	↑(Kidd et al., 2007) ↓(Chen et al., 2015)		↑(Jia Q. et al., 2017)	↓(Ji et al., 2015)	↑(Fang et al., 2014) ↓(Lee et al., 2016)
Glioma	↑(Xie et al., 2004a)	↑(Xie et al., 2004b)	↓(Gupta et al., 2001) ↑(Laurent et al., 2003)	↑(Tao et al., 2020)	↑(Minchenko et al., 2015)	
Head and neck cancer	↑(Liu et al., 2015)	↑(Wu et al., 2017)				
Kidney cancer			↓(Liu et al., 2012)	↑(Xu et al., 2000)		
Leukemia	↑(Niu et al., 2014)	↑(Wells et al., 2016)	↓(McCallum et al., 2012)	↑(Zhang X. et al., 2015)		
Liver cancer	↑(Li et al., 2018) ↓(Feng et al., 2008)	↑(Jia S. et al., 2017; Makino et al., 2018) ↓(Isbert et al., 2007)	↑(Jia Q. et al., 2017)	↑(Chen et al., 2018) ↓(Zhang H. et al., 2015)	↑(Chen Z. et al., 2016)	↓(Gao et al., 2019)
Lung cancer	↓(Tong et al., 2001)	↓(Chang et al., 2004, 2013)		↑(Matsubara et al., 2005) ↓(Soon et al., 2003)		
Ovarian cancer	↑(Gery et al., 2005)	↓(Barbolina et al., 2009)		↑(Graumann et al., 2019)		
Pancreatic cancer	↑(Haque et al., 2011; Maity et al., 2014)	↑(Bennewith et al., 2009)	↑(Cui et al., 2014)	↑(Yang et al., 2015)	↑(Wang et al., 2013)	
Prostate cancer	↑(Sun et al., 2008) ↓(D’Antonio et al., 2010)	↑(Yang et al., 2005)	↑(Chen et al., 2014)	↑(Tai et al., 2014) ↓(Ono et al., 2013)		
Melanoma		↓(Chen J. et al., 2016)		↑(Deng et al., 2019)		
Salivary gland tumors	↑(Lai et al., 2014)			↑(Lencioni et al., 2016)	↓(Kouzu et al., 2006)	
Oral squamous cell carcinoma	↑(Kok et al., 2010)	↓(Chuang et al., 2011)		↑(Jung et al., 2017; Chang et al., 2019)		
Endometrial cancer	↓(Chien et al., 2004)					
Laryngeal cancer				↑(Wang et al., 2019)		

**The numbers in brackets are reference numbers. ↑ CCN acting as tumor promotor; ↓CCN acting as tumor suppressor.**

### Expression and Roles of CCN1 in Pan-Cancer

**FIGURE 3:**
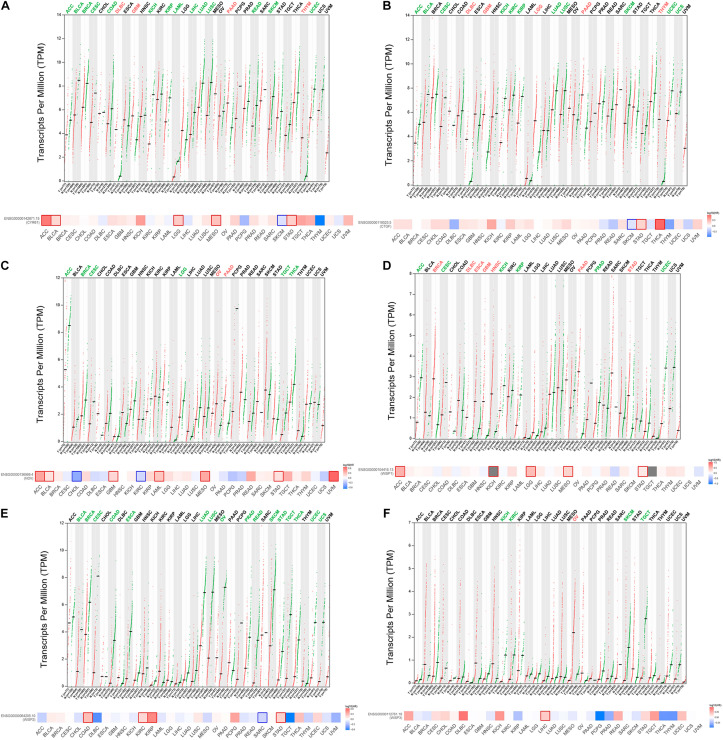
mRNA levels of CCN1-6 differ between human pan-cancer and normal tissue, suggesting their potential role as prognostic and therapeutic biomarkers. **(A)** CCN1 expression and association with OS. **(B)** CCN2 expression and association with OS. **(C)** CCN3 expression and association with OS. **(D)** CCN4 expression and association with OS. **(E)** CCN5 expression and association with OS. **(F)** CCN6 expression and association with OS. For gene expression profile dot plot, color-coded cancers’ abbreviation suggests significant results (*p* < 0.05) and red mean gene over expressed in cancer tissue compared with the normal tissue, while green have reversed meaning. For survival heat map, blocks with border suggest significant results (*p* < 0.05) and red blocks mean high expression of CCNs has a poor prognosis, while blue blocks have reversed meaning.

CCN1 exhibits varying mRNA levels and associations with prognosis across different types of cancer. Comparisons of CCN1 mRNA levels among 32 human cancer types and adjacent normal tissue using GEPIA2 revealed significantly upregulated CCN1 expression in four types of cancer [lymphoid neoplasm diffuse large B-cell lymphoma (DLBC), glioblastoma multiforme (GBM), pancreatic adenocarcinoma (PAAD), and thymoma (THYM)] and significantly downregulated expression in 14 types of cancer [adrenocortical carcinoma (ACC), bladder urothelial carcinoma (BLCA), breast invasive carcinoma (BRCA), cervical squamous cell carcinoma and endocervical adenocarcinoma (CESC), colon adenocarcinoma (COAD), kidney chromophobe (KICH), kidney renal papillary cell carcinoma (KIRP), acute myeloid leukemia (LAML), liver hepatocellular carcinoma (LIHC), lung adenocarcinoma (LUAD), lung squamous cell carcinoma (LUSC), rectum adenocarcinoma (READ), skin cutaneous melanoma (SKCM), and uterine corpus endometrial carcinoma (UCEC)]. To evaluate the association between CCN1 mRNA expression and prognosis, we also examined 32 human cancers using GEPIA2. The relationship between CCN1 expression and prognosis varied across different types of cancer. High expression of CCN1 was associated with shorter overall survival (OS) in five types of cancer [ACC, BLCA, brain lower grade glioma (LGG), mesothelioma (MESO), and stomach adenocarcinoma (STAD)] and longer OS only in SKCM, suggesting its role as a tumor suppressor. These bioinformatics results revealed the heterogeneous expression and functions of CCN1 in different types of cancer (Figure 3A).

Several previous studies reported that CCN1 participates in cancer development and can serve as both a tumor suppressor and promoter (Barreto et al., 2016). In most types of cancer, CCN1 acts as an oncogene (Tan et al., 2009; Xie et al., 2011, 2019; Niu et al., 2014; Liu et al., 2015; Su et al., 2019; Khandelwal et al., 2020). By contrast, in esophageal (Dang et al., 2018), liver (Feng et al., 2008), prostate (D’Antonio et al., 2010), lung (Tong et al., 2001), and endometrial (Chien et al., 2004) cancer, CCN1 serves as a protective role. Mori et al. reported that CCN1 mRNA level is lower in lung cancer tissue than in normal lung tissue (Mori et al., 2007), consistent with our bioinformatics results. Also, Tong et al. (2001) showed that overexpression of CCN1 in non-small cell lung cancer cell lines reduces colony formation and proliferation, thus serving as a tumor suppressor. As summarized in Table 1, previous, mostly *in vitro*, studies showed that CCN1 serves as a tumor promoter in most cancers but can also acts as a tumor suppressor in some cancers. Thus, to resolve the discrepant roles of CCN1 in different types of cancer, future studies should take diverse TMEs and different isoforms into consideration.

### Expression and Roles of CCN2 in Pan-Cancer

CCN2 mRNA levels and their association with prognosis also vary across different types of cancer. Comparison of CCN2 mRNA levels among different cancer tissues and their adjacent normal tissues revealed significantly higher CCN2 expression in five types of cancer (DLBC, GBM, LGG, PAAD, and THYM) and significantly lower expression in 11 types of cancer [ACC, BLCA, CESC, KICH, KIRC, KIRP, LUAD, LUSC, SKCM, esophageal carcinoma, and uterine carcinosarcoma (UCS)]. When we evaluated associations between CCN2 mRNA levels and prognosis, we found that high expression of CCN2 was associated with shorter OS in STAD and THCA and longer OS only in SKCM, suggesting that it acts as a tumor suppressor. Thus, these bioinformatics results further revealed the heterogeneous expression and function of CCN2 in different types of cancer (Figure 3B).

After reviewing the current studies on CCN proteins. In gastric cancer, high CCN2 expression correlates with more lymph node metastases, more peritoneal dissemination, and poorer 5-year survival (Cheng et al., 2014). After CCN2 downregulation, gastric cancer cells show attenuated migratory/invasive abilities and decreased protein expression of MMPs (Jiang et al., 2011). Recently, Pamrevlumab (FG-3019), a first-in-class antibody that inhibits the activity of CCN2, received fast-track designation from the U.S. Food and Drug Administration for the treatment of patients with idiopathic pulmonary fibrosis and locally advanced unresectable pancreatic cancer (Ramazani et al., 2018). CCN2 overexpression is related to poor prognosis in most types of cancer (Chien et al., 2004). Even so, there have been plenty of opposite reports in gastrointestinal cancer (Chen et al., 2015), liver cancer (Isbert et al., 2007), lung cancer (Chang et al., 2013), ovarian cancer (Barbolina et al., 2009), and melanoma (Chen J. et al., 2016). Table 1 summarizes the functional roles of CCN2 across different types of cancer.

### Expression and Roles of CCN3 in Pan-Cancer

Comparison of CCN3 mRNA levels among different types of cancer tissues and their adjacent normal tissues revealed that CCN3 expression was significantly upregulated in two types of cancer [ovarian serous cystadenocarcinoma (OV) and PAAD] and significantly downregulated in six types of cancer [ACC, BRCA, CESC, LGG, testicular germ cell tumors (TGCT), and thyroid carcinoma (THCA)]. When we further evaluated the association between CCN3 expression and prognosis in pan-cancer, we found that high CCN3 expression was associated with shorter OS in seven types of cancer (ACC, BLCA, GBM, LAML, MESO, STAD, and uveal melanoma) and longer OS in two types of cancer (CHOL and KIRC, Figure 3C).

CCN3 was first discovered as an overexpressed gene in myeloblastosis-associated virus type-1-induced nephroblastoma (Joliot et al., 1992) and has since been implicated in many diverse biological processes, such as proliferation, differentiation, angiogenesis and fibrosis, all of which promote cancer development (Barreto et al., 2016). CCN3 has anti-tumor effects in breast cancer (Dobson et al., 2014), colorectal tumors (Li et al., 2017), kidney cancer (Liu et al., 2012), glioma (Gupta et al., 2001), and leukemia (McCallum et al., 2012). By contrast, CCN3 acts as a tumor promoter in liver (Jia Q. et al., 2017), pancreatic (Cui et al., 2014), and prostate (Chen et al., 2014) cancer. Laurent et al. (2003) reported that in glioma, CCN3 triggers a cascade of gene expression resulting in increased cell adhesion and migration. Our group showed that CCN3 is a hallmark in the development and chemoresistance of liver cancer (Holbourn et al., 2009; Perbal and Perbal, 2016) via regulation of cell stemness and the TME (Holbourn et al., 2009; Tang et al., 2019). Table 1 provides a summary of CCN3 expression and functional roles in different types of cancer, and the heterogeneous roles of CCN3 are also revealed in different types of cancer.

### Expression and Roles of CCN4 in Pan-Cancer

Similar to other CCN family members, CCN4 mRNA levels and their association with prognosis vary across different types of cancer. Comparison of CCN4 mRNA levels among diverse cancer types and adjacent normal tissue revealed significantly higher CCN4 expression in seven types of cancer (BRCA, DLBC, ESCA, GBM, HNSC, PAAD, and STAD). When evaluating the association between CCN4 expression and prognosis in pan-cancer, we found that high CCN4 expression was associated with shorter OS in five types of cancer (ACC, KICH, LGG, MESO, STAD). The results of these bioinformatics analyses suggest that CCN4 mainly acts as a tumor promoter (Figure 3D).

The participation of CCN4 in cancer development has been reported by many previous studies, which showed that CCN4 serves as a tumor promoter in colorectal (Wu et al., 2016), breast (Xie et al., 2001b), pancreatic (Yang et al., 2015), and lung (Chen et al., 2007) cancer by enhancing cell migration and promoting epithelial-mesenchymal transition (EMT). However, in breast (Taghavi et al., 2016), lung (Soon et al., 2003), and liver (Zhang H. et al., 2015) cancer, CCN4 appears to play an opposing role. Davies et al. (2007) showed that CCN4 acts as a tumor suppressor in breast cancer based on examination of mRNA levels in human breast tumor tissues compared with normal tissues. Tao et al. (2020) showed that CCN4 plays dual roles in glioblastoma—both maintaining glioma stem cells and constructing a pro-TME via the infiltration of tumor-supportive macrophages. Zhang X. et al. (2015) found reduced CCN4 expression in liver tumors compared with normal liver tissue, suggesting that CCN4 serves as a tumor suppressor. CCN4 expression is regulated by various signaling pathways and is sensitive to different biochemical perturbations in the TME, which may explain its diverse roles in cancer progression. Table 1 provides a summary of CCN4 expression and its functional roles in different types of cancer.

### Expression and Roles of CCN5 in Pan-Cancer

CCN5 mRNA levels also vary across different types of cancer. Comparison of CCN5 mRNA levels across different cancer types and adjacent normal tissue revealed significantly lower expression of CCN5 in 16 types of cancer (BLCA, BRCA, CESC, COAD, ESCA, LUAD, LUSC, OV, PRAD, READ, SKCM, SATD, TGCT, THCA, UCEC, and UCS). Increased expression of CCN5 was not observed in any type of cancer. High CCN5 expression was associated with shorter OS in four types of cancer (COAD, KIRC, KIRP, and STAD) and longer OS only in SARC, suggesting that CCN5 acts as an anti-oncogene. The results of these bioinformatics analyses suggest that CCN5 expression and function vary across different types of cancer, perhaps due to differences in its structure compared with other CCN family members (Figure 3E).

As CCN5 lacks a CT domain, this striking difference in structure compared with other CCN family members may allow it to have unique functional roles. Like its family members, however, previous studies reported inconsistent roles of CCN5 in carcinogenesis. CCN5 is downregulated in human leiomyoma (Mason et al., 2004), pancreatic adenocarcinoma (Dhar et al., 2007), salivary gland cancer (Dhar et al., 2007), colorectal tumors (Pennica et al., 1998; Davies et al., 2010), and gallbladder cancer (Yang et al., 2014), suggesting that it acts as a tumor suppressor. Chai et al. (2019) showed that CCN5 overexpression inhibits cell growth, induces apoptosis, and suppresses cell migration and invasion in esophageal squamous cell carcinoma. Banerjee et al. (2008) showed that the expression of CCN5 is undetectable in normal breast tissues but increased in non-invasive breast cancer lesions, suggesting that it acts as a negative regulator of migration and invasion. By contrast, in glioma (Minchenko et al., 2015), liver cancer (Chen Z. et al., 2016), and pancreatic cancer (Wang et al., 2013), CCN5 acts as a tumor promoter. Whereas CCN5 mainly localizes in the nucleus in human cancer tissue (Wiesman et al., 2010), we found that CCN5 is expressed in both the cytoplasm and nucleus in malignant kidney tumors, with predominate cytoplasmic expression (unpublished data). Table 1 summarizes the expression and diverse roles of CCN5 across different types of cancer.

### Expression and Roles of CCN6 in Pan-Cancer

CCN6 mRNA levels and prognostic value also vary depending on the type of cancer. Comparison of CCN6 mRNA levels among diverse cancer types and adjacent normal tissue revealed that CCN6 expression was significantly downregulated in four types of cancer (KICH, KIRC, SKCM, and TGCT) and significantly upregulated only in OV. When evaluating the association between CCN6 expression and prognosis, we found that high CCN6 expression was associated with shorter OS only in LIHC. These bioinformatics analyses further suggest that the expression and functions of CCN6 are inconsistent across cancer types (Figure 3F).

CCN6 has received much attention in the last few years due to its involvement in many cancer-related processes, including EMT, cell death, invasion, and metastasis, and its function as a tumor suppressor (Tran and Kleer, 2018). However, many studies reported that CCN6 can serve as both a tumor suppressor and promoter (Lee et al., 2016). CCN6 is expressed in normal breast epithelium but is reduced or lost in 60% of invasive breast carcinomas (Huang et al., 2008). CCN6 limits breast cancer invasion and metastasis by modulating the BMP signaling pathway (Pal et al., 2012). By contrast, CCN6 is overexpressed in 63% of human colon tumors and appears to be associated with colon tumorigenesis (Pennica et al., 1998). In addition, CCN6 is related to microsatellite instability in colorectal cancer (Thorstensen et al., 2001). As summarized in Table 1, the studies showed expression and functional roles of CCN6 are also inconsistent among different types of cancer.

## Conclusion and Perspectives

The six members of CCN proteins have established associations with cancer malignancy progression and are considered as prognostic markers and therapeutic targets for several types of cancer. However, CCN proteins always appear to have contradictory roles in different types of cancer. After a retrospective analysis of the literature, we come to the conclusions (Arbyn et al., 2020). Cellular locations, tissue specificity of CCN proteins expression and the diverse TME provide some explanation for their apparently conflicting functions (Winkler et al., 2020). The presence of multiple functional domains of CCN proteins and the altered biological activity of truncated CCN proteins increasing the diversity of CCNs biological roles in cancer (Jiang et al., 2019). CCN protein functions could be orchestrated by other CCN members, and the final biological properties of a specific CCN protein might be dependent on the combinations of CCN members.

Targeting CCN protein expression or signaling pathways holds promise in the development of diagnostics and therapeutics for cancers, and the cocktail containing CCN proteins in different combinations should be a potential antitumor approach. Since the current literature has certain limitations in clarifying the exact role of CCN proteins, continued studies are still needed to reveal the exact roles of CCN proteins in cancer.

## Author Contributions

QJ contributed to the conceptualization, literature search, writing, review, and editing. BX contributed to the literature search and editing. YZ contributed to the methodology and visualization. AA contributed to language proofreading. XL contributed to the critical review and editing. All authors have read and approved the final manuscript.

## Conflict of Interest

The authors declare that the research was conducted in the absence of any commercial or financial relationships that could be construed as a potential conflict of interest.

## Abbreviations

Cancer types: ACC: adrenocortical carcinoma
AML: acute myeloid leukemia
BLCA: bladder urothelial carcinoma
BRCA: breast invasive carcinoma
CESC: cervical and endocervical cancers
CHOL: cholangiocarcinoma
COAD: colon adenocarcinoma
DLBC: lymphoid neoplasm diffuse large B-cell lymphoma
ESCA: esophageal carcinoma
GBM: Glioblastoma multiforme
HNSC: head and neck squamous cell carcinoma
KICH: kidney chromophobe
KIRC: kidney renal clear cell carcinoma
KIRP: Kidney renal papillary cell carcinoma
AML: Acute Myeloid Leukemia
LGG: brain lower grade glioma
LIHC: liver hepatocellular carcinoma
LUAD: lung adenocarcinoma
MESO: mesothelioma
OV: ovarian serous cystadenocarcinoma
PAAD: pancreatic adenocarcinoma
PCPG: pheochromocytoma and paraganglioma
PRAD: prostate adenocarcinoma
SARC: sarcoma
SKCM: skin cutaneous melanoma
STAD: stomach adenocarcinoma
TGCT: testicular germ cell tumors
THCA: thyroid carcinoma
THYM: thymoma
UCEC: uterine corpus endometrial carcinoma
UVM: uveal melanoma
TME: tumor microenvironment
CCN: cellular communication network
CCN1/CYR61: cysteine-rich 61
CCN2/CTGF: connective tissue growth factor
CCN3/NOV: nephroblastoma overexpressed
CCN4/WISP-1: Wnt1-inducible signaling pathway proteins 1
CCN4/WISP-2: Wnt1-inducible signaling pathway proteins 2
CCN4/WISP-3: Wnt1-inducible signaling pathway proteins 3
TCGA: The Cancer Genome Atlas
IGFBP: insulin-like growth factor-binding protein
VWC: Von Willebrand factor type C
BMPs: bone morphogenic proteins
TSP-1: The thrombospondin type 1 repeat
VEGF: vascular endothelial growth factor
HSPGs: heparan sulfate proteoglycans
CT: carboxyterminal
EMT: epithelial-mesenchymal transition.
